# Interactions between mitochondrial reactive oxygen species and cellular glucose metabolism

**DOI:** 10.1007/s00204-015-1520-y

**Published:** 2015-06-06

**Authors:** Dania C. Liemburg-Apers, Peter H. G. M. Willems, Werner J. H. Koopman, Sander Grefte

**Affiliations:** Department of Biochemistry (286), Radboud Institute for Molecular Life Sciences (RIMLS), Radboud University Medical Center (RUMC), P.O. Box 9101, 6500 HB Nijmegen, The Netherlands; Department of Human and Animal Physiology, Wageningen University, P.O. Box 338, 6700 AH Wageningen, The Netherlands

**Keywords:** Glucose, GLUT1, GLUT4, Mitochondria, ROS, Oxidative stress

## Abstract

Mitochondrial reactive oxygen species (ROS) production and detoxification are tightly balanced. Shifting this balance enables ROS to activate intracellular signaling and/or induce cellular damage and cell death. Increased mitochondrial ROS production is observed in a number of pathological conditions characterized by mitochondrial dysfunction. One important hallmark of these diseases is enhanced glycolytic activity and low or impaired oxidative phosphorylation. This suggests that ROS is involved in glycolysis (dys)regulation and vice versa. Here we focus on the bidirectional link between ROS and the regulation of glucose metabolism. To this end, we provide a basic introduction into mitochondrial energy metabolism, ROS generation and redox homeostasis. Next, we discuss the interactions between cellular glucose metabolism and ROS. ROS-stimulated cellular glucose uptake can stimulate both ROS production and scavenging. When glucose-stimulated ROS production, leading to further glucose uptake, is not adequately counterbalanced by (glucose-stimulated) ROS scavenging systems, a toxic cycle is triggered, ultimately leading to cell death. Here we inventoried the various cellular regulatory mechanisms and negative feedback loops that prevent this cycle from occurring. It is concluded that more insight in these processes is required to understand why they are (un)able to prevent excessive ROS production during various pathological conditions in humans.

## Introduction

Mitochondria are among the prime ATP-generating organelles, which are necessary for cellular functioning (Koopman et al. 2012, 2013). To this end, glucose is oxidized to pyruvate in the cytosol by the glycolysis pathway. Next, pyruvate enters the mitochondria where it is converted into acetyl-coenzyme-A that is further oxidized within the tricarboxylic acid (TCA) cycle. Alternatively, acetyl-coenzyme-A can be generated by fatty acid breakdown during β-oxidation. Conversion of acetyl-coenzyme-A by the TCA cycle yields reduced nicotinamide adenine dinucleotide (NADH) and reduced flavin adenine dinucleotide (FADH_2_). These two molecules serve as electron donors for mitochondrial complex I (CI or NADH:ubiquinone oxidoreductase) and complex II (CII or succinate:ubiquinone oxidoreductase) of the electron transport chain (ETC). The electrons are subsequently transported by ubiquinone to complex III (CIII or ubiquinol:cytochrome-*c* oxidoreductase) and by cytochrome-*c* to complex IV (CIV or cytochrome-*c* oxidase) where they react with oxygen to form water. At CI, CIII and CIV protons are expelled from the mitochondrial matrix across the mitochondrial inner membrane (MIM). This results in establishment of an inward-directed proton motive force (PMF) that consists of a chemical (ΔpH) and electrical (Δψ) component (Mitchell 1961). Via complex V (CV or F_1_F_o_-ATP synthase), protons are allowed to flow back into the matrix to fuel generation of ATP from ADP and inorganic phosphate (P_i_). Together with the ETC, CV constitutes the mitochondrial oxidative phosphorylation (OXPHOS) system.

### Mitochondrial ROS generation

Both as a consequence of normal electron transport and during mitochondrial dysfunction, electrons can escape from the ETC to induce formation of superoxide anions by one-electron reduction of oxygen. This means that, under certain conditions, mitochondria can substantially contribute to the generation of cellular reactive oxygen species (ROS; Adam-Vizi and Chinopoulos 2006; Murphy 2009). Interestingly, several proteins involved in glycolysis, mitochondrial electron transport, β-oxidation and the TCA cycle are also able to generate superoxide, hydrogen peroxide and/or other ROS. These include CI (Grivennikova and Vinogradov 2013; Murphy 2009; Treberg et al. 2011), CII (Quinlan et al. 2012a; Siebels and Drose 2013), CIII (Muller et al. 2004; Murphy 2009), dihydroorotate dehydrogenase (DHOH; Forman and Kennedy 1975; Orr et al. 2012), pyruvate dehydrogenase (PDH; Fisher-Wellman et al. 2013; Starkov et al. 2004), aconitase (Gardner 2002; Vasquez-Vivar et al. 2000), 2-oxoglutarate dehydrogenase (Odh, or α-ketoglutarate dehydrogenase; Bunik and Sievers 2002; Quinlan et al. 2014; Starkov et al. 2004; Tretter and Adam-Vizi 2004) and Sn-glycerol-3-phosphate dehydrogenase (mGPDH; Orr et al. 2012). In addition, various other mitochondrial proteins like monoamine oxidases (MAOs) and p66shc/cytochrome-*c* (Di Lisa et al. 2009; Giorgio et al. 2005; Hauptmann et al. 1996) are capable of ROS production. Regarding the ETC, CI and CIII are the most well characterized (Murphy 2009). In case of CI, superoxide production can occur at two sites: the flavin mononucleotide (FMN) site and the iron–sulfur cluster (Genova et al. 2001; Herrero and Barja 2000; Johnson et al. 2003; Kussmaul and Hirst 2006; Lambert and Brand 2004; Treberg et al. 2011). Alternatively, hydrogen peroxide might be directly formed at the FMN site (Grivennikova and Vinogradov 2013). In CIII, evidence was provided that superoxide is produced only at the quinol-oxidizing (Q_O_) site (Kramer et al. 2004; Muller et al. 2003; Murphy 2009). Inhibitor studies suggested that superoxide and/or hydrogen peroxide can also be produced at the flavin site of CII (Quinlan et al. 2012a).

However, in these studies, the exact sites and magnitude of ROS production depend on the used OXPHOS substrates and inhibitors, respectively. In the absence of inhibitors, (native) ROS production appears to be much lower (Quinlan et al. 2012b, 2013). Since these studies use isolated mitochondria, the situation might also be different in intact cells and tissues.

### Maintaining redox homeostasis

To prevent unintentional generation of redox signals and induction of oxidative stress, mitochondria possess powerful antioxidant systems. One of these consists of manganese-dependent superoxide dismutase (MnSOD or SOD2), an enzyme that is localized in the mitochondrial matrix and rapidly converts superoxide to hydrogen peroxide. This conversion is also catalyzed by the copper/zinc-dependent superoxide dismutase (Cu/ZnSOD or SOD1), which is localized in the cytosol, nucleus and mitochondrial intermembrane space (Murphy 2009; Tyler 1975; Weisiger and Fridovich 1973). In turn, hydrogen peroxide can be converted into water by the action of catalases that are mainly located in the peroxisomes and also in mitochondria (Salvi et al. 2007). However, within mitochondria, hydrogen peroxide is mainly removed by the action of glutathione peroxidase-1 (Gpx1; Cox et al. 2010; Esposito et al. 2000; Esworthy et al. 1997), peroxiredoxins 3 and 5 (Prx3 and Prx5) and the thioredoxin-2 (Trx2) system (Chae et al. 1999; Chang et al. 2004; Cox et al. 2010), which require glutathione (GSH). Oxidized GSH (GSSG) is recycled to GSH by the action of glutathione reductase. Similarly, oxidized Trx2 is recycled by Trx reductase. Both of these systems require NADPH (Arner 2009; Carlberg and Mannervik 1985), which is regenerated in the cytosol by glucose-6-phosphate-dehydrogenase (G6PDH) via the pentose phosphate pathway (PPP; Le Goffe et al. 2002). Alternatively, NADPH can be regenerated in the mitochondrial matrix by nicotinamide nucleotide transhydrogenase (NNT), which uses NADH and the PMF (Hatefi and Yamaguchi 1996; Rydstrom 2006; Yin et al. 2012).

### ROS signaling

ROS have the ability to modulate the transcription and activity of enzymes, receptors and transporter (Mailloux et al. 2014; Martinez-Reyes and Cuezva 2014; Sena and Chandel 2012), for instance during adaptation to exercise (Gomez-Cabrera et al. 2005; Silveira et al. 2006). To fulfill a signaling function, ROS should be able to induce reversible protein modifications, thereby affecting its activity and/or function. Hydrogen peroxide is believed to be a main player in ROS signaling due to its physicochemical properties, which include a relatively low reactivity, long half-life and the ability to diffuse through membranes (Forman et al. 2010; Winterbourn and Hampton 2008). Mechanistically, hydrogen peroxide can oxidize thiol groups (–SH) on exposed cysteine residues in proteins, resulting in the formation of sulfenic acid (–SO^−^, known as S-oxidation or sulfenylation) (Carballal et al. 2003; Charles et al. 2007; Seres et al. 1996). Subsequently, the sulfenic acid group can: (1) form inter- and intramolecular disulfide bonds with other thiol groups leading to altered protein structure or the formation of homo- and/or heterodimers (Brennan et al. 2004; Delaunay et al. 2002; Rehder and Borges 2010; Yang et al. 2007), (2) react with GSH (–SSG; thereby inducing S-glutathionylation of the protein; (Chen et al. 2007b; Hurd et al. 2008; McLain et al. 2013), or (3) react with amides to form a sulfenyl amide (Salmeen et al. 2003; Sivaramakrishnan et al. 2010). Although not discussed in this review, mitochondria are also exposed to various reactive nitrogen species (RNS), which can induce oxidative protein modifications (Beltran et al. 2000; Boveris et al. 2006; Hogg 2002; Rossig et al. 1999; Zaobornyj and Ghafourifar 2012). Interestingly, most of the above modifications are reversible (reviewed in detail elsewhere: Forman et al. 2010; Handy and Loscalzo 2012; Mailloux et al. 2014), i.e., disulfide bonds between thiol groups can be reduced by the Trx reductase system, while glutathionylated thiol groups can be reduced by glutaredoxin (Grx2) utilizing the GSH pool (Beer et al. 2004; Handy and Loscalzo 2012; Mailloux et al. 2014). Regarding ROS regulation of protein activity, several phosphatases contain thiol groups in their active site, which upon oxidation lead to a loss of dephosphorylation activity (Rhee et al. 2000; Tonks 2005). Also, proteins of the TCA cycle and OXPHOS are regulated by ROS (Mailloux et al. 2014). For example, S-glutathionylation has been shown to reduce the activity of CI (Hurd et al. 2008) and Odh (McLain et al. 2013).

### Oxidative stress induction

When mitochondrial ROS production exceeds the capacity of the cell’s antioxidant systems or when the latter systems are less active, increased ROS levels can induce cell damage (oxidative stress). In principle, superoxide can react with protein iron–sulfur (Fe–S) clusters (Liochev and Fridovich 1994), which, in the presence of hydrogen peroxide, induce generation of hydroxyl radicals (Fenton reaction). The latter are highly reactive and can damage lipids, proteins and DNA (Martinez-Reyes and Cuezva 2014; Valko et al. 2006). Given the fact that iron is effectively sequestered (Kakhlon and Cabantchik 2002), the relevance of the Fenton reaction is not fully established in vivo. Superoxide can react with NO to form peroxynitrite (ONOO^−^; Pacher et al. 2005). When rising too high, hydrogen peroxide can induce over-oxidization of cysteine residues from sulfenic acid (–SO^−^) to sulfinic acid (–SO_2_H) and sulfonic acid (–SO_3_H). In case of CI, such over-oxidation is associated with irreversible deactivation of CI (Hurd et al. 2008; Mailloux et al. 2014). In general, if ROS levels exceed a certain threshold, they will impair OXPHOS complexes and further stimulate ROS production (Galloway and Yoon 2012). In the light of the above, it is not surprising that increased ROS levels, although not always oxidative stress, are observed during various pathological conditions. For example, primary fibroblasts derived either from CI deficient mice or patients show increased ROS levels, but no obvious signs of oxidative stress (Koopman et al. 2007; Valsecchi et al. 2013; Verkaart et al. 2007a, b). Increased ROS levels also have been observed in multiple types of cancer (e.g., prostate, colorectal, ovarian, pancreatic, breast, liver, bladder, melanoma, glioma), neurogenerative diseases (e.g., Alzheimer’s disease and Parkinson’s disease) and during insulin-resistance and diabetes (Afanas’ev 2011; Freeman et al. 2006; Kumar et al. 2008; Pi and Collins 2010; Sabens Liedhegner et al. 2012; Sanchez-Gomez et al. 2013). Below we will discuss the interplay between ROS levels, glucose uptake and metabolism in detail.

## Regulation of glucose uptake by reactive oxygen species

Stimulation of cellular glucose uptake is frequently observed during conditions of oxidative stress. Exogenous addition of hydrogen peroxide stimulates glucose uptake in skeletal muscle (Higaki et al. 2008; Jensen et al. 2008; Kim et al. 2006), C2C12 myoblasts, clone 9 liver cells and 3T3 fibroblasts (Prasad and Ismail-Beigi 1999). Upon electrical stimulation, endogenous ROS also induced an increase in glucose uptake in muscle cells (Merry et al. 2010; Pinheiro et al. 2010). Interestingly, in L6 myoblasts, inhibition of cellular glucose uptake was associated with increased ROS levels (Andrisse et al. 2014), perhaps suggesting a role for glucose in ROS scavenging. Cellular glucose uptake is mediated by glucose transporters (GLUTs), of which fourteen isoforms have been described with different kinetic properties and modes of regulation (Carruthers et al. 2009; Joost and Thorens 2001). Here we will primarily focus on GLUT1 and GLUT4, which are abundantly expressed in muscle cells and well studied. GLUT1 is expressed during all stages of embryonic development (Hogan et al. 1991). After birth, GLUT1 expression decreases, but most cell types still express low levels of GLUT1 to mediate basal glucose uptake. However, GLUT1 expression remains high in cells that primarily depend on glycolysis for ATP generation such as erythrocytes and tumor cells. In the latter, GLUT1 is frequently up-regulated (Baer et al. 1997; Brown and Wahl 1993; Nishioka et al. 1992), which is associated with poor survival in various malignant tumors (Szablewski 2013). GLUTs are expressed in a highly tissue-specific manner (Bell et al. 1990; Gould and Holman 1993). This, in combination with the fact that the different GLUTs display different functional characteristics, allows for a tissue-specific regulation of glucose uptake (Gould and Holman 1993). For example, GLUT4 expression is up-regulated in differentiating muscle cells (Mitsumoto and Klip 1992), which show increased levels of OXPHOS complexes and higher respiration rates (Mitsumoto and Klip 1992; Remels et al. 2010). This suggests that under certain conditions (i.e., differentiation), cells (co)express both GLUT1 and other GLUTs to facilitate increased glucose uptake to support increased cellular respiration. In fact, under basal conditions, the majority of GLUT4 in muscle cells is retained in intracellular vesicles that are derived from the trans-Golgi network. Retention of these vesicles requires the activity of the Rab GTPase activating proteins TBC1D1 and TBC1D4 (Eguez et al. 2005; Larance et al. 2005; Sano et al. 2003). Stimulation by insulin or exercise induces translocation of these vesicles to the plasma membrane. Although the full signaling cascade regulating vesicle translocation is still incompletely understood, Akt-mediated inhibition of TBC1D1/TBC1D4 plays an essential role (Eguez et al. 2005; Funaki et al. 2004; Kohn et al. 1998; Larance et al. 2005; Ng et al. 2008; Sano et al. 2003). It appears that following translocation to the plasma membrane, an additional activation step is required for stimulation of glucose uptake (Funaki et al. 2004; Somwar et al. 2002; Sweeney et al. 1999).

### Transcriptional regulation of GLUT expression by ROS

Protein expression levels of human GLUT1 are controlled by a promoter region and several putative enhancer regions that contain binding sites for various transcription factors including specificity proteins (Sp1; Vinals et al. 1997) and hypoxia-inducible factor-1 (HIF-1; Ebert et al. 1995). Mild oxidative stress induced by either glucose/glucose oxidase (Glc/GO) or xanthine/xanthine oxidase (Xan/XO) has been shown to up-regulate GLUT1 expression by increasing the transcription rate and mRNA stability leading to increased GLUT1 protein and glucose transport activity (Kozlovsky et al. 1997). As far as we know, there is no experimental evidence demonstrating the involvement of Sp1 in ROS-induced stimulation of GLUT1 expression, and therefore, we here focus on the role of HIF-1.

**Fig. 1:**
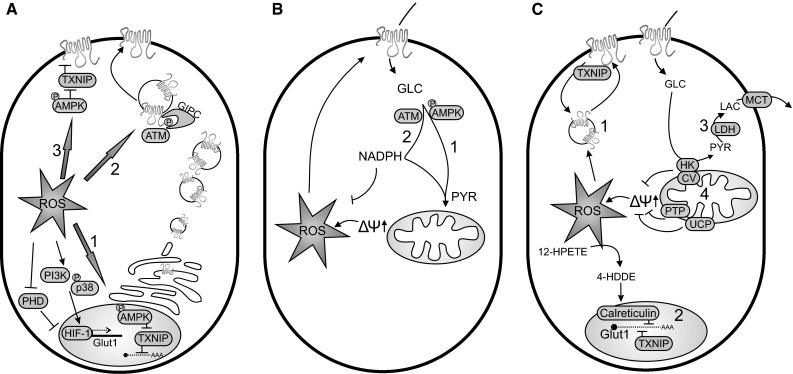
Interplay between ROS and glucose. **a** Glucose uptake can be regulated by: (*1*) altering the expression level of glucose transporters (GLUTs; *blue*), (*2*) stimulating translocation of GLUTs from internal vesicles to the plasma membrane and (*3*) changing the intrinsic activity of GLUTs at the plasma membrane. **b** Glycolytic conversion of glucose into pyruvate and subsequent pyruvate entry into the mitochondria (*1*) stimulates ROS production by hyperpolarizing the mitochondrial membrane potential (Δψ↑). Subsequently, ROS stimulate glucose uptake (see **a**), thereby triggering additional ROS production. Glucose flux through the pentose phosphate pathway (stimulated by AMPK and ATM) generates NADPH (*2*), which is an important cofactor in ROS scavenging. **c** Hyperpolarization of the mitochondrial membrane potential (Δψ↑) is prevented by: (*1*) GLUT1 internalization, (*2*) GLUT1 mRNA degradation, (*3*) reduction of pyruvate to lactate and subsequent secretion of lactate. A hyperpolarized mitochondrial membrane potential is diminished by: (*4*) transient uncoupling of the mitochondrial membrane potential (PTP, UCP) or enhancing oxidative phosphorylation efficiency by HK–CV interaction. Proteins that are activated by ROS are depicted in yellow (for details, see main text). 4-*HDDE* 4-hydroxydodecadienal, 12-*HPETE* 12-hydroperoxyeicosatetraenoic acid, Δψ mitochondrial membrane potential, *ATM* ataxia telangiectasia mutated, *CV* complex V, *GIPC* Gα-interacting protein-interacting protein, C-terminus, *GLC* glucose, *Glut1* glucose transporter 1, *HIF*-1 hypoxia-inducible factor 1, *HK* hexokinase, *LAC* lactate, *LDH* lactate dehydrogenase, *MCT* monocarboxylate transporter, *P*-*AMPK* phosphorylated (activated) AMP-activated protein kinase, *PHD* prolyl hydroxylase domain, *P*-p38, phosphorylated (activated) p38 mitogen-activated protein kinase, PI3K phosphoinositide 3-kinase, *PTP* permeability transition pore, *PYR* pyruvate, *ROS* reactive oxygen species, *TXNIP* thioredoxin-interacting protein, *UCP* uncoupling protein (color figure online)

HIF-1 consists of two subunits, HIF-1α and HIF-1β. Under normoxic conditions, prolines within the oxygen-dependent degradation domains (ODDs) of HIF-1α are hydroxylated by prolyl-4-hydroxylases (PHDs; Ivan et al. 2001). This hydroxylation acts as an ubiquitination signal leading to proteasomal degradation of HIF-1α. In the absence of oxygen, HIF-1α ubiquitinylation is inhibited allowing its interaction with HIF-1β to drive transcription of various target genes, including GLUT1 (Hayashi et al. 2004; Iyer et al. 1998; Ouiddir et al. 1999; Wood et al. 1998). During hypoxia, ROS levels increase and play an important role in HIF-1α stabilization (Brunelle et al. 2005; Chandel et al. 2000; Guzy et al. 2005; Mansfield et al. 2005; Sanjuan-Pla et al. 2005; Schroedl et al. 2002). Preventing ROS-mediated HIF-1α stabilization represses GLUT1 expression and glucose uptake in Lewis lung carcinoma, HT-29 colon, and T47D breast cancer cells (Jung et al. 2013). Upon mitochondrial DNA depletion (Chandel et al. 2000; Mansfield et al. 2005) and in mouse embryonic fibroblasts (MEFs) lacking cytochrome-*c* (Mansfield et al. 2005), hypoxia-induced HIF-1α stabilization is abrogated. This suggests that hypoxia-induced ROS are of mitochondrial origin. Knockout of the Rieske iron–sulfur protein (RISP) in mitochondrial CIII decreases ROS production during hypoxia and attenuates hypoxic stabilization of HIF-1α (Guzy et al. 2005). Therefore, RISP-mediated mitochondrial ROS production appears to be involved in HIF-1α stabilization during hypoxia. At the RISP site, electrons are transferred one-by-one from ubiquinol to cytochrome-*c*1. This one-electron donation generates a highly reactive ubisemiquinone, which can act as a source for superoxide generation. Over-expression of catalase (Chandel et al. 2000; Guzy et al. 2005) or GPx1 (Brunelle et al. 2005; Emerling et al. 2005) abolishes HIF-1α stabilization during hypoxia, whereas over-expression of SOD1 or SOD2 does not (Brunelle et al. 2005; Guzy et al. 2005). In addition, exogenous hydrogen peroxide is sufficient to stabilize HIF-1α under normoxic conditions (Chandel et al. 2000; Jung et al. 2008; Mansfield et al. 2005). This suggests that the stabilization of HIF-1α primarily involves hydrogen peroxide via inactivation of PHDs (Fig. 1a) and subsequent reduction of HIF-1α ubiquitinylation (Chandel et al. 1998; Guzy and Schumacker 2006). However, HIF-1α ubiquitinylation is incompletely blocked by exogenous or hypoxia-derived hydrogen peroxide (Guzy et al. 2005), suggesting the involvement of additional mechanisms.

Alternatively, ROS-induced HIF-1α stabilization was proposed to be mediated by specific signaling routes. Phosphoinositide 3-kinase (PI3K) appears to be involved in HIF-1α stabilization by mitochondrial ROS in Hep3B cells (Fig. 1a; arrow 1) (Chandel et al. 2000). Compatible with this mechanisms, PI3K inhibition lowered GLUT1 transcription under hypoxic and normoxic conditions (Chen et al. 2001). In addition, both p38 mitogen-activated protein kinase (MAPK p38) and its upstream kinases MKK3/6 are activated by hypoxia-derived ROS and are essential in HIF-1α stabilization and the ensuing increase in GLUT1 mRNA levels (Emerling et al. 2005). Activation of MAPK p38 by ROS is mediated by apoptosis signal-regulating kinase 1 (ASK1), which is normally bound to Trx. Oxidation of Trx releases ASK1 leading to activation of its downstream targets MKK3, MKK4, MKK6 and MMK7 and subsequently JNK and MAPK p38 (Nagai et al. 2007). Also, AMP-activated protein kinase (AMPK) can be activated by ROS during hypoxia (Emerling et al. 2009). This protein plays a key role in energy metabolism and cellular adaptation to ROS (Wu et al. 2014). Various metabolic stress conditions can activate AMPK through phosphorylation of Thr172 by upstream kinases such as LKB1 (Hardie 2011). In addition, AMP-interaction stimulates AMPK activation by promoting AMPK phosphorylation, preventing AMPK dephosphorylation and inducing allosteric activation of AMPK (Davies et al. 1995; Hardie and Ashford 2014). Oxidative stress can inhibit the ETC, potentially leading to an increased AMP:ATP ratio (Hawley et al. 2010). However, under hypoxic conditions, AMPK is also activated by mitochondrial ROS in an LKB1-dependent and AMP:ATP-independent manner (Emerling et al. 2009; Mungai et al. 2011). In muscle cells, AMPK activation stimulated GLUT1 expression and twofold increased cellular glucose uptake (Fryer et al. 2002). Besides activation of HIF-1α, AMPK can up-regulate GLUT1 via degradation of thioredoxin-interacting protein (TXNIP) (Fig. 1a; arrow 1). TXNIP reduces the level of GLUT1 mRNA in the nucleus. TXNIP is degraded upon Ser308 phosphorylation by AMPK, leading to increased GLUT1 mRNA and protein levels (Wu et al. 2013).

### Regulation of GLUT-vesicle translocation to the plasma membrane by ROS

The cellular capacity for glucose uptake is co-determined by the abundance of functional GLUTs at the plasma membrane. This means that glucose uptake can be regulated by GLUT trafficking between the cytosol and the plasma membrane. During exercise, muscle contraction is associated with increased ROS levels, believed to represent a fast adaptive response to an increase in energy demand (Chambers et al. 2009; Murrant et al. 1999; Wretman et al. 2001). In these cells, endogenous (i.e., those induced by contraction) and exogenous ROS stimulate glucose uptake via a mechanism involving activation of Akt and/or AMPK (Higaki et al. 2008; Sandstrom et al. 2006; Toyoda et al. 2004). ROS can activate Akt (Fernandes et al. 2011; Niwa et al. 2003), which probably involves inactivation of cysteine-based phosphatases (Okoh et al. 2011). ROS can activate AMPK by modification of cysteine residues on its α-catalytical subunit (Zmijewski et al. 2010). Once activated, Akt and AMPK phosphorylate TBC1D4 and TBC1D1, leading to GLUT4 translocation (Geraghty et al. 2007; Kramer et al. 2006; Taylor et al. 2008; Thong et al. 2007).

Translocation of GLUT1 to the plasma membrane (Fig. 1a; arrow 2) is regulated by Ataxia telangiectasia mutated (ATM; Andrisse et al. 2013). This protein is a member of the family of phosphatidylinositol-3-kinase-related protein kinases (PIKKs) and plays an important role in the response to DNA damage after which it becomes phosphorylated. However, ATM can also localize to the cytosol where it is activated by ROS and involved in cytosolic signaling (Alexander and Walker 2010). ATM is thiol-oxidized by ROS to form an active dimer of two covalently linked monomers (Guo et al. 2010). Activated ATM localizes near mitochondria and mitochondria are necessary for ROS-induced ATM activation (Morita et al. 2014). This suggests that ATM dimerization and activation are stimulated by mitochondrial ROS. Evidence was provided that ATM is activated by ROS during treatment with the chemotherapeutic agent doxorubicin (Kurz et al. 2004). In muscle, ATM activation by doxorubicin mediates targeting of GLUT1 to the cell surface by phosphorylation of GLUT1 at S490. This serine residue is part of a C-terminal PDZ motif, and phosphorylation of this residue induces interaction of GLUT1 with the PDZ-interacting protein, Gα-interacting protein and C-terminus (GIPC1) (Andrisse et al. 2013). GIPC1 interaction promotes GLUT1 trafficking to the cell surface and stimulates glucose uptake (Fig. 1a; arrow 2) (Wieman et al. 2009).

### Regulation of GLUT1 activity at the plasma membrane by ROS

Regulation of glucose uptake can also occur directly at the plasma membrane. This type of regulation occurs on a relatively short-term time scale (i.e., within 1 h) and represents a relatively fast mechanism that allows the cells to cope with metabolic stress (Fig. 1a; arrow 3). The intrinsic activity of GLUTs can be modulated by conformational changes or posttranslational modifications that increase glucose affinity (Asano et al. 1991; Levine et al. 2002). In addition, the maximal rate of cellular glucose uptake (V_max_) can be modulated by activation or deactivation of GLUTs at the plasma membrane. A number of metabolic inhibitors and AMPK activators acutely stimulate glucose uptake without increasing the amount of GLUT molecules at the plasma membrane (Abbud et al. 2000; Barnes et al. 2002; Hamrahian et al. 1999; Shetty et al. 1993; Shi et al. 1995). It was proposed that this increase is due to the release of “masking proteins,” which display an inhibitory interaction with the GLUT cytoplasmic domain under basal conditions (Shi et al. 1995). Stomatin was proposed being a masking protein as it interacts with the C-terminus of GLUT1, and its over-expression reduces GLUT1 intrinsic activity (Rungaldier et al. 2013; Zhang et al. 2001). Another potential masking protein is TXNIP, which displays an inhibitory interaction with GLUT1 (Wu et al. 2013). AMPK-induced degradation of TXNIP (see previous section) would unmask GLUT1 leading to enhancement of GLUT1-mediated glucose influx (Fig. 1a; arrow 3). Although stimulation of GLUT intrinsic activity is often observed during metabolic stress, the role of ROS in this pathway is still incompletely understood. In a leukemic cell line, ROS generated by NAD(P)H oxidase (Nox) stimulated glucose uptake via Src-mediated phosphorylation of GLUT1 (Prata et al. 2008). On the other hand, antioxidants were ineffective in preventing GLUT1 activation during azide-induced CIV inhibition (Hamrahian et al. 1999). Taken together, various signaling pathways have been implied in the stimulation of glucose uptake as an adaptive response during oxidative stress (Fig. 1a). At a relatively slow timescale, ROS-induced signals stimulate glucose uptake via up-regulation of GLUT protein expression. Rapid stimulation of glucose uptake can occur at the level of GLUT translocation and regulation of GLUT intrinsic activity. Interestingly, several of the signaling proteins are ROS-sensitive (i.e., AMPK and TXNIP) and involved in both slow and fast responses. This suggests that ROS-induced stimulation of glucose uptake is part of a (adaptive) mechanism triggered by oxidative and/or metabolic stress.

## Effects of increased glucose uptake on ROS production and scavenging

The above evidence supports the conclusion that increased ROS levels stimulate cellular glucose uptake both at slow and fast time scales. However, inhibition of GLUT1 activity in myoblasts was paralleled by increased ROS levels (Andrisse et al. 2014). This might indicate that glucose uptake also plays a role in regulating the balance between ROS production and scavenging, suggesting that glucose uptake must be tightly controlled to maintain cellular energy homeostasis and redox status.

### The role of glucose in ROS scavenging

Glucose entry into the PPP is protective against hydrogen peroxide-induced cytotoxicity (Le Goffe et al. 2002). Mechanistically, this protection is probably due to an increased PPP flux, leading to a higher NADPH/NADP^+^ ratio and GSH level. Compatible with this hypothesis, skin fibroblasts derived from patients with MERFF (myoclonic epilepsy with ragged red fibers) displayed GLUT1 up-regulation and increased NADPH and GSH levels (Wu and Wei 2012). Preventing this NADPH increase induced ROS over-production and cell death (Wu and Wei 2012). Glucose entry into the PPP and subsequently increased levels of NADPH is stimulated by ATM-induced activation of G6PDH (Fig. 1b; arrow 2), which is the first and rate determining enzyme of the PPP (Cosentino et al. 2011). Moreover, GLUT1 contributes to ROS scavenging by mediating the transport of dehydroascorbic acid (DHA), the oxidized form of vitamin C, which is recycled back to vitamin C inside the cell (Rumsey et al. 1997). Vitamin C is a potent antioxidant, which can prevent oxidative cell death (De Rosa et al. 2010; Guaiquil et al. 2001). Experimental evidence suggests that GLUT1 also co-localizes with mitochondria to facilitate mitochondrial uptake of DHA and quench ROS induced by mitochondrial uncoupling (Kc et al. 2005). In summary, enhanced PPP glucose entry and GLUT-mediated antioxidant uptake appear to be relevant for cellular ROS removal.

### The role of glucose in stimulation of ROS production

A high glycolytic flux was associated with increased ROS levels (Talior et al. 2003; Zhou et al. 2005), whereas inhibition of mitochondrial pyruvate uptake lowered ROS levels (Nishikawa et al. 2000; Yu et al. 2006). Increased glycolytic flux is generally associated with and increased TCA cycle flux (Ishihara et al. 1996). The latter results in accumulation of OXPHOS substrates and elevated NADH/NAD^+^ and FADH_2_/FAD ratios (Ido 2007; Ying 2008). A high level of mitochondrial NADH leads to a fully reduced FMN site in CI, potentially stimulating superoxide production (Kussmaul and Hirst 2006). Similarly, a more reduced ETC might stimulate superoxide formation by CIII (Turrens et al. 1985). Reduced ubiquinone, in combination with a highly negative (hyperpolarized) Δψ, favors reverse electron transfer from CII to CI, which also stimulates superoxide production (Batandier et al. 2006; Murphy 2009). Hyperglycemic conditions induced mitochondrial fragmentation in clone 9 liver cells, H9c2 cardiomyoblasts, and smooth muscle cells, which was strictly required to allow increased ROS production (Yu et al. 2011). However, induction of mitochondrial fragmentation by over-expression of the fission-promoting protein Drp1 (Dynamin-related protein 1), did not stimulate ROS levels in HeLa cells, suggesting that increased ROS levels are not a *de facto* consequence of mitochondrial fragmentation (Distelmaier et al. 2012). It was proposed that fragmented mitochondria might produce more ROS due to a bigger relative membrane surface (allowing better uptake of metabolic substrates) and ensuing Δψ hyperpolarization (Yu et al. 2006). High extracellular glucose levels stimulate TXNIP expression (Stoltzman et al. 2008), which can aggravate oxidative stress by binding Trx via disulfide bridges and thereby inhibiting its reducing potential (Hwang et al. 2014; Kaimul et al. 2007; Li et al. 2015; Nishiyama et al. 1999; Schulze et al. 2004). Taken together, the current experimental evidence suggests that increased glucose uptake and glycolytic conversion to pyruvate can increase ROS levels.

## Cellular and metabolic adaptation to increased ROS levels

Integrating the above mechanisms, it is conceivable that certain (pathological) conditions favor activation of a self-amplifying cycle of glucose uptake and glucose-stimulated ROS production, ultimately leading to cell death. Glucose-stimulated ROS production might be counterbalanced by the combined action of endogenous antioxidant systems (Sect. 1.2) and glucose-stimulated increase in ROS scavenging (Sect. 3.1). If these systems are insufficient, cells might also prevent glucose-induced oxidative stress by other means. As discussed in Sect. 3.2, a very high glycolytic flux eventually results in increased ETC electron input, a highly negative (hyperpolarized) Δψ, and a (probably) more reduced ETC. These phenomena all favor mitochondrial ROS production by the ETC. As discussed in the following sections, several mechanisms have been described that reduce ETC-mediated ROS generation. The latter generally are associated with a less reduced state of CI and CIII, likely associated with reduced electron leak and ROS generation, and include lowering the amount of electrons fed into the ETC and induction of (partial) Δψ depolarization.

### Reducing the ETC electron input

In principle, ETC electron input can be reduced by lowering cellular glucose uptake. A high extracellular glucose concentration triggers a rapid reduction in GLUT1 levels at the plasma membrane, without affecting their total cellular levels (Greco-Perotto et al. 1992; Sasson et al. 1997). Such a reduction can be induced by inhibition of GLUT translocation to the plasma membrane, stimulation of GLUT internalization or both (Fig. 1c; process 1). On a slower time scale, down-regulation of GLUT expression (Fig. 1c; process 2) also lowers the total and plasma membrane levels of GLUT (Riahi et al. 2010; Totary-Jain et al. 2005). Regulation of these two processes is mediated by various signaling pathways. First, a high rate of glucose uptake induces the buildup of glycolytic intermediates, which activate the transcription factor carbohydrate response element-binding protein (chREBP) to drive the expression of a number of target genes, including TXNIP, that negatively regulate glycolysis (Stoltzman et al. 2008). TXNIP over-expression inhibits glucose uptake, while TXNIP knockdown stimulates glucose uptake (Parikh et al. 2007). TXNIP inhibits glucose uptake by interacting with GLUT1 and possibly inducing GLUT1 internalization through clathrin-coated pits (Fig. 1c; process 1). In addition, TXNIP reduces glucose uptake by suppressing GLUT1 mRNA levels (Fig. 1c; process 2; Wu et al. 2013). A TXNIP mutant unable to bind Trx still inhibited glucose uptake (Parikh et al. 2007; Patwari et al. 2009), suggesting that TXNIP-induced down-regulation of glucose uptake does not exclusively depend on Trx (and possibly redox status). Besides having a stimulatory effect on glucose uptake, ROS also exert a negative feedback on glucose uptake. For instance, exogenous application of ROS induced GLUT1 internalization and reduced glucose uptake (Fig. 1c; process 1) in retinal endothelial cells (Fernandes et al. 2004, 2011). Similarly, a prolonged exposure to exogenous ROS triggered Akt inactivation and GLUT1 internalization (Fernandes et al. 2011). In contrast, a short exposure to exogenous ROS stimulated Akt activation and translocation of GLUT1 to the plasma membrane (Fernandes et al. 2011). The above suggests a mechanism in which ROS first transiently stimulates glucose uptake (perhaps to increase NADPH levels) and subsequently down-regulates glucose uptake to prevent that too much electrons are fed into the ETC. However, internalization of GLUT1 does not always occur efficiently and high glucose-induced ROS production is prolonged (Cohen et al. 2007; Rosa et al. 2009). The exact reason for the absence of GLUT1 reduction at the plasma membrane is not well understood. It is possible that regulatory proteins such as Akt are bypassed. It may also be a consequence of irreversible damage (induced by ROS) of regulatory proteins, such as those comprising the proteasome machinery. In muscle, increased ROS levels during enhanced glucose uptake reduce the level of GLUT1 mRNA by increasing the expression of 12-lipoxygenase. This enzyme converts arachidonic acid to 12-hydroperoxyeicosatetraenoic acid (12-HPETE) (Alpert et al. 2002) and ROS-induced oxidation of the latter leads to formation of 4-hydroxydodecadienal (4-HDDE). This molecule activates the peroxisome proliferator-activated receptor δ (PPARδ) to drive the expression of calreticulin (Riahi et al. 2010). On its turn, calreticulin interacts with a cis-acting element in the 3′UTR of GLUT1 mRNA, making it susceptible to degradation (Totary-Jain et al. 2005) (Fig. 1c; process 2).

Inhibition of lactate dehydrogenase (LDH) enhanced oxidative stress and cell death in tumor cells (Le et al. 2010), whereas stimulating pyruvate-to-lactate conversion reduced oxidative stress (Brand 1997). This suggests that ETC electron input is reduced by stimulating conversion of pyruvate into lactate and secretion of the latter by the cell into the extracellular environment (Fig. 1c; process 3). Another mechanism to reduced ETC electron input is shown by the fact that ROS can stimulate glutathionylation or sulfenation, and thereby inactivation, of several TCA cycle enzymes including PDH, KGDHC, aconitase, isocitrate dehydrogenase, and CII (Bulteau et al. 2005; Chen et al. 2007b; Kil and Park 2005; McLain et al. 2011, 2013; Yan et al. 2013). Although ROS-induced inhibition of the TCA cycle results in a decline of NADH and in turn diminish the mitochondrial electron feed and hence reduced state of CI, it may also be detrimental to cells (Tretter and Adam-Vizi 2000). Increased ROS levels can also reduce the glycolytic flux by activation of poly(ADP-ribose) polymerase (PARP), leading to subsequent inactivation of glyceraldehyde-3-phosphate dehydrogenase (GAPDH) (Du et al. 2003; Nishikawa et al. 2000). Although this mechanism might be useful to lower ETC electron input, it also makes glucose enter the polyol pathway, which consumes NADPH and generates NADH (Giacco and Brownlee 2010). This is unfavorable since NADPH depletion and increased NADH/NAD^+^ ratios are associated with increased ROS production and/or levels.

### Depolarization of Δψ

The magnitude of Δψ is mainly determined by the gradient of protons across the MIM, but also other charged ions and small molecules (e.g., ATP^3−^, ADP^4−^) can play a role. Therefore, every mechanism that reduces proton influx or increases proton efflux into the MIM, when not counterbalanced, stimulates (partial) Δψ depolarization. In this sense, Δψ depolarization can be induced by ETC inhibition, CV stimulation (increased coupling efficiency) and/or increased trans-MIM proton leak (uncoupling). The latter is mediated by mitochondrial uncoupling proteins (UCPs) 1-3 (Fig. 1c; process 4, UCP). In mitochondrial fractions from various tissues, GDP-induced inhibition of UCP1 and UCP2 was associated with Δψ hyperpolarization and increased production of hydrogen peroxide (Negre-Salvayre et al. 1997). Similarly, in muscle mitochondria and intact muscle fibers inhibition of UCP3 by GDP-stimulated superoxide and hydrogen peroxide production, respectively (Anderson et al. 2007; Talbot et al. 2004). Also genetic intervention studies revealed that absence of either UCP2 or UCP3 resulted in higher ROS production and oxidative stress in mitochondria and cells (Anderson et al. 2007; Arsenijevic et al. 2000; Brand et al. 2002; Lee et al. 2009; McLeod et al. 2005; Seifert et al. 2008; Toime and Brand 2010; Valsecchi et al. 2013). In line with these results, UCP2 or UCP3 over-expression lowered mitochondrial ROS production (Lee et al. 2005; Teshima et al. 2003; Valsecchi et al. 2013). Surprisingly, ROS can activate both UCP2 and UCP3 via deglutathionylation rather than glutathionylation, although the latter is normally stimulated by high ROS levels (Mailloux et al. 2011). In this sense, high levels of ROS activate UCPs and thereby constituting a negative feedback loop that lowers mitochondrial ROS production presumably via mitochondrial uncoupling and ensuing Δψ depolarization. In addition to the role of UCPs in controlling mitochondrial ROS production via Δψ depolarization, also a role for these proteins in metabolism has been proposed (Bouillaud 2009; Huppertz et al. 2001). This suggests that UCP-mediated metabolic changes might (co)determine mitochondrial ROS generation.

Fast Δψ depolarization can also be induced by opening of the mitochondrial permeability transition pore (PTP) (Fig. 1; process 4, PTP). Although the mitochondrial matrix protein Cyclophilin D (CypD) is a well-characterized regulator of PTP opening, the exact molecular composition of this channel remains elusive. Genetic intervention studies revealed that the adenine nucleotide translocase (ANT) and the mitochondrial phosphate carrier (PiC) are not core components but also regulators of PTP opening (Gutierrez-Aguilar et al. 2014; Kokoszka et al. 2004; Kwong et al. 2014; Varanyuwatana and Halestrap 2012). More recently, a central role for mitochondrial CV has been proposed (Alavian et al. 2014; Bonora et al. 2013; Giorgio et al. 2013). However, it may well be that an interaction of the CV with ANT and PiC is the mechanism of PTP formation (Halestrap 2014). Most of the above proteins have been shown to be prone to ROS-induced modification. The ANT contains several thiol residues that are sensitive to redox modification and involved in regulating PTP opening (Costantini et al. 2000; McStay et al. 2002; Queiroga et al. 2010). Upon diamide-induced oxidative stress, Cys^160^ cross-links to Cys^257^ thereby locking the ANT in its “c-conformation,” which sensitizes the PTP to elevated calcium levels within the mitochondrial matrix (McStay et al. 2002). Carbon monoxide is able to induce glutathionylation of ANT and decreasing PTP opening, while in turn diamide is able to deglutathionylate ANT (Martinez-Reyes and Cuezva 2014). Cys^203^ of CypD is another target for oxidative modification, shown to be involved in ROS-induced PTP opening and cell death. Upon Cys^203^ mutation, PTP opening is reduced to a level similar to that in CypD-negative cells (Linard et al. 2009; Nguyen et al. 2011). The α-subunit of the CV can also be glutathionylated leading to a decrease in CV activity (Garcia et al. 2010). Whether this decrease reduces PTP opening still needs to be elucidated. Although PTP opening might reduce the mitochondrial ROS levels by lowering ROS production and/or allow the release of mitochondrial ROS into the cytosol, this phenomenon has also been associated with induction of superoxide “flashes” (Wang et al. 2008; Zhang et al. 2013). However, the existence and chemical nature of these flashes is currently debated as the biosensor used for superoxide detection also appears to be pH-sensitive (Muller 2009; Schwarzlander et al. 2012, 2014). More importantly, sustained PTP opening triggers apoptosis and has been linked to pathophysiology and cell death (Brenner and Moulin 2012; Ichas and Mazat 1998). This suggests that only short reversible PTP openings, associated with reversible Δψ depolarizations (Blanchet et al. 2014), are suited to reduce mitochondrial ROS levels and preserve cell viability.

### Other mechanisms that reduce ROS

Increased coupling efficiency of the OXPHOS system (i.e., between ETC electron transport and CV-mediated ATP production) has also been described as a mechanism to depolarize Δψ and lower ROS production (Fig. 1; process 4, HK/CV) (Starkov and Fiskum 2003). Recruitment of hexokinase (HK) to the outer surface of the mitochondrial outer membrane (MOM) was associated with decreased hydrogen peroxide production (Sun et al. 2008). A mechanism was proposed in which HK uses OXPHOS-derived ATP to metabolize glucose and form ADP. The latter is exchanged with ATP across the MIM by the ANT and used to fuel ATP production by CV. In principle, such a mechanism would increase coupled respiration and thereby reduce ETC electron leak and ROS production. However, glucose-6-phosphate accumulation is stimulated under conditions of increased glycolytic flux. This accumulation might inhibit HK activity and thereby increase mitochondrial ROS production (da-Silva et al. 2004). As increased HK-mediated ADP cycling enhances ETC electron transport, this mechanism should also result in a less reduced state of the FMN site of CI and thereby might decrease mitochondrial ROS formation. The latter could also be achieved by lowering NADH production (Sect. 4.1) or by decreasing OXPHOS activity. In case of CII and CV, their glutathionylation has been associated with reduced activity (Chen et al. 2007b, 2008; Garcia et al. 2010). CI also contains subunits that are sensitive to oxidative modification. These include the 51-kDa (NDUFV1) subunit (Cys^187^, Cys^206^, Cys^425)^ and 75-kDa (NDUFS1) subunit (Cys^367^ Cys^531^ Cys^704^ Cys^226^ Cys^727^), which can be glutathionylated and thereby diminish the activity of CI (Beer et al. 2004; Chen et al. 2007a; Hurd et al. 2008; Kang et al. 2012). NDUFV1 and NDUFS1 glutathionylation is reversible and protects CI from further oxidative damage such as sulfenylation and thereby irreversible deactivation (Hurd et al. 2008). Compatible with inhibitor studies (Koopman et al. 2010), CI inhibition by glutathionylation was linked to increased superoxide production (Taylor et al. 2003). This suggests that CI inactivation is not necessarily associated with reduced mitochondrial ROS production. Taken together, the above suggests that oxidative stress triggered by increased glucose uptake could trigger adaptive responses to reduce mitochondrial ROS production via reducing ETC electron input, depolarization of Δψ or increasing coupled respiration.

## Summary and conclusion

ROS are produced as a consequence of normal mitochondrial energy metabolism. When transiently and/or moderately increased, ROS can activate signaling pathways involved in cellular adaptation to various types of (metabolic) stress. One of these pathways is the stimulation of glucose uptake. When ROS levels are too high and/or remain increased during a prolonged period of time, a vicious circle of ROS-stimulated glucose uptake and glucose-stimulated ROS production can be triggered. This pathological cycle can be broken by restoring mitochondrial ROS production to normal levels. We presented three major mechanisms that, in principle, can lower mitochondrial ROS production: (1) reducing glucose uptake, (2) increasing lactate secretion and (3) depolarization of Δψ. Unfortunately, these mechanisms have also been associated with increases in ROS and/or appear to be not effective in all experimental models. Undesirable side effects include reduced NADPH production during reduced glucose uptake, a high rate of lactate secretion potentially inducing lactic acidosis and induction of mitochondrial dysfunction and apoptosis by (high-magnitude) and/or prolonged Δψ depolarization. We conclude that cellular glucose metabolism and mitochondrial ROS production are coupled by various signaling mechanisms, which need to be controlled by the cell to avoid oxidative stress. A more detailed understanding of how these pathways interact with mitochondrial ROS production, endogenous antioxidant systems and mitochondrial/cellular function is required to explain why oxidative stress induction still appears to contribute to pathology induction in humans (e.g., diabetes, cancer, mitochondrial dysfunction).

## Abbreviations

Δψ: Mitochondrial membrane potential
4-HDDE: 4-Hydroxydodecadienal
12-HPETE: 12-Hydroperoxyeicosatetraenoic acid
ASK1: Apoptosis signal-regulating kinase 1
AMPK: AMP-activated protein kinase
ANT: Adenine nucleotide translocator
ATM: Ataxia telangiectasia mutated
CI: Complex I or NADH: ubiquinone oxidoreductase
CII: Complex II or succinate:ubiquinone oxidoreductase
CIII: Complex III or ubiquinol:cytochrome-c oxidoreductase
CIV: Complex IV or cytochrome-c oxidase
Cu/ZnSOD: Copper/zinc-dependent superoxide dismutase or SOD1
CV: Complex V or F_1_F_o_-ATP synthase
chREBP: Carbohydrate response element-binding protein
CypD: Cyclophilin D
ETC: Electron transport chain
DHA: Dehydroascorbic acid
DHOH: Dihydroorotate dehydrogenase
FADH_2_: Reduced flavin adenine dinucleotide
FMN: Flavin mononucleotide
G6PDH: Glucose-6-phosphate-dehydrogenase
GAPDH: Glyceraldehyde-3-phosphate dehydrogenase
GIPC1: Gα-interacting protein-interacting protein, C-terminus
Glc/GO: Glucose/glucose oxidase
GLUT: Glucose transporter
Gpx: Glutathione peroxidase
Grx2: Glutaredoxin
GSH: Glutathione
GSSG: Oxidized glutathione
HIF-1: Hypoxia-inducible factor-1
HK: Hexokinase
LDH: Lactate dehydrogenase
MAOs: Monoamine oxidases
MAPK p38: p38 mitogen-activated protein kinase
MEFs: Mouse embryonic fibroblasts
mGPDH: Sn-glycerol-3-phosphate dehydrogenase
MIM: Mitochondrial inner membrane
MnSOD: Manganese-dependent superoxide dismutase or SOD2
MOM: Mitochondrial outer membrane
PiC: Inorganic phosphate carrier
PTP: Permeability transition pore
NADH: Reduced nicotinamide adenine dinucleotide
NNT: Nicotinamide nucleotide transhydrogenase
Nox: NAD(P)H oxidase
ODDs: Oxygen-dependent degradation domains
Odh: 2-Oxoglutarate dehydrogenase
OXPHOS: Oxidative phosphorylation
PARP: Poly(ADP-ribose) polymerase
PDH: Pyruvate dehydrogenase
PHDs: Prolyl-4-hydroxylases
P_i_: Inorganic phosphate
PI3K: Phosphoinositide 3-kinase
PIKK: Phosphatidylinositol-3-kinase-related protein kinase
PMF: Proton motive force
PPARδ: Peroxisome proliferator-activated receptor δ
PPP: Pentose phosphate pathway
Prx: Peroxiredoxins
RISP: Rieske iron–sulfur protein
ROS: Reactive oxygen species
SOD1: Copper/zinc-dependent superoxide dismutase or CuZnSOD
SOD2: Manganese-dependent superoxide dismutase or MnSOD
Sp1: Specificity protein
TCA: Tricarboxylic acid
Trx: Thioredoxin
TXNIP: Thioredoxin-interacting protein
UCP: Uncoupling protein
Xan/XO: Xanthine/xanthine oxidase
